# Cardiac Amyloidosis: A Review of Current Imaging Techniques

**DOI:** 10.3389/fcvm.2021.751293

**Published:** 2021-12-10

**Authors:** Yousuf Razvi, Rishi K. Patel, Marianna Fontana, Julian D. Gillmore

**Affiliations:** National Amyloidosis Centre, Division of Medicine, University College London, London, United Kingdom

**Keywords:** cardiac amyloid, CMR (cardiovascular magnetic resonance), echo, ATTR, light chain (AL) amyloidosis, hATTR, patisiran, inotersen

## Abstract

Systemic amyloidosis is a rare, heterogenous group of diseases characterized by extracellular infiltration and deposition of amyloid fibrils. Cardiac amyloidosis (CA) occurs when these fibrils deposit within the myocardium. Untreated, this inevitably leads to progressive heart failure and fatality. Historically, treatment has remained supportive, however, there are now targeted disease-modifying therapeutics available to patients with CA. Advances in echocardiography, cardiac magnetic resonance (CMR) and repurposed bone scintigraphy have led to a surge in diagnoses of CA and diagnosis at an earlier stage of the disease natural history. CMR has inherent advantages in tissue characterization which has allowed us to better understand the pathological disease process behind CA. Combined with specialist assessment and repurposed bone scintigraphy, diagnosis of CA can be made without the need for invasive histology in a significant proportion of patients. With existing targeted therapeutics, and novel agents being developed, understanding these imaging modalities is crucial to achieving early diagnosis for patients with CA. This will allow for early treatment intervention, accurate monitoring of disease course over time, and thereby improve the length and quality of life of patients with a disease that historically had an extremely poor prognosis. In this review, we discuss key radiological features of CA, focusing on the two most common types; immunoglobulin light chain (AL) and transthyretin (ATTR) CA. We highlight recent advances in imaging techniques particularly in respect of their clinical application and utility in diagnosis of CA as well as for tracking disease change over time.

## Introduction

Systemic amyloidosis encompasses a rare, heterogenous group of diseases caused by extracellular deposition of amyloid fibrils in the tissues which are identified by apple green birefringence when stained with Congo red dye *ex vivo* and viewed under cross polarized light. This insoluble fibrillar material is derived from a variety of normally soluble precursor proteins which misfold and self-assemble with abnormal cross beta-sheet conformation that is typically stable and resistant to proteolysis (1, 2). More than 30 human precursor proteins have been identified which can form amyloid fibrils *in vivo*, and amyloidosis is classified accordingly. However, whilst amyloid fibrils are derived from a variety of structurally different precursor proteins, they retain a shared core structure that is relatively stable and resistant to proteolysis (2, 3).

Disease is caused once the accumulation of amyloid fibrils is sufficient to disrupt the inherent structure and function of the affected organ (1). Amyloid deposition can occur in almost any organ of the body; cardiac amyloid deposition inevitably results in a restrictive and/or infiltrative cardiomyopathy. Whilst systemic amyloidosis is a multi-organ disease, cardiac amyloidosis remains the leading cause of mortality (4, 5). Two precursor proteins are responsible for the majority of cases of CA, namely monoclonal immunoglobulin light chains in AL amyloidosis and transthyretin in ATTR amyloidosis. AL amyloidosis arises as a consequence of a clonal proliferation of plasma cells or B cells which may, in itself, be very subtle. Treatment of AL amyloidosis is based on chemotherapy to eradicate ongoing production of amyloidogenic monoclonal light chains paired with supportive management such as diuretic therapy to manage heart failure (6). Transthyretin, a circulating protein which is synthesized in the liver and is responsible for the transport of thyroxine and retinol binding protein, may form amyloid when unmutated with advancing age (wild-type ATTR amyloidosis) or in association with over 130 genetic variants (5, 6). Wild-type ATTR amyloidosis (wtATTR) manifests as a predominant cardiomyopathy (ATTR-CM) whilst variant (ATTRv) or hereditary (hATTR) ATTR amyloidosis is typically associated with polyneuropathy (ATTR-PN) as well as cardiomyopathy (4). In the absence of specific disease-modifying therapy, ATTR-CM is inexorably progressive and ultimately fatal; clinical management until recently was limited to supportive management of heart failure with diuretics and rhythm control (4, 5).

Studies have shown that ATTR-CM has been widely underdiagnosed, with one large UK study indicating a median of 17 hospital attendances per patient before the correct diagnosis was established, and 42% of patients waiting over 4 years from the onset of cardiac symptoms to diagnosis (7). However, recent advances in diagnostic imaging techniques involving advanced echocardiography, cardiac magnetic resonance (CMR) imaging and repurposed bone scintigraphy have allowed the diagnosis of ATTR-CM to be established more easily and more widely without a need for demonstration of amyloid on histology in ~70% of cases (8). The same imaging techniques also play an important role in the accurate diagnosis of AL cardiomyopathy (AL-CM) (6, 8), and have also contributed to a substantial increase in recent years of this diagnosis (7). These advances in diagnostic imaging have been accompanied by landmark developments in therapy including novel disease-modifying therapeutics for ATTR amyloidosis and better chemotherapeutic agents for AL amyloidosis (6, 9–11). Together, the advances in imaging and therapy have transformed the care of patients with CA in recent years.

## AL Cardiac Amyloidosis

AL amyloidosis has long been considered the most common form of systemic amyloidosis, with an approximate prevalence of 10 per million people per year (1). Cardiac amyloid infiltration is present in ~80% of cases and is the main driver of morbidity and mortality (12). AL cardiac amyloidosis (AL-CM) confers a poorer prognosis and is associated with a more aggressive clinical course than ATTR-CM, despite the fact that the cardiac amyloid burden and left ventricular (LV) mass in ATTR-CM usually exceeds that in AL-CM (6). AL amyloid infiltrates all cardiac chambers with biventricular thickening leading to restrictive cardiomyopathy and cardiac failure is common. Complicating conduction disease resulting in significant bradyarrhythmia can also be a feature (5, 6). A recent study demonstrated that myocardial oedema was present by CMR imaging and by histology in untreated AL-CM when compared to ATTR and treated AL cardiac amyloidosis, consistent with the idea of light chain toxicity and related mechanisms contributing to myocardial damage. The presence of oedema was also significantly associated with a poor prognosis (5, 13).

Whilst AL-CM remains the leading cause of mortality in AL amyloidosis, extra-cardiac manifestations are common; AL amyloid can infiltrate almost any organ in the body. This results in a heterogeneous and often non-specific disease presentation with consequent diagnostic delay (2, 4). However, ~30% of cases present with pathognomonic clinical signs which include macroglossia and peri-orbital bruising (1, 2). Typical initial clinical manifestations of AL-CM include dyspnoea, weight loss and fatigue. The dyspnoea usually has a relatively rapid onset and progression and is often accompanied by peripheral oedema and/or ascites, which may not only be due to severe right sided cardiac failure, but also contributed to by hypoalbuminaemia secondary to renal amyloid infiltration resulting in heavy proteinuria (3, 6). It is worth noting, that presentation with clear cardiac symptoms may not be evident until the cardiac disease is advanced (4).

Chemotherapy in AL amyloidosis, the mainstay of disease-modifying therapy, is aimed at suppressing ongoing production of amyloidogenic light chains by targeting the underlying clonal dyscrasia, usually originating from plasma cells. Despite the recent availability of novel agents which achieve higher rates and speed of clonal suppression with fewer toxicities, overall prognosis remains notably poor among those with advanced AL-CM at diagnosis, compounded by the diagnostic delays highlighted above (14–16). Early diagnosis, achieved through use of the correct imaging modalities, is therefore key to improving patient outcomes in this otherwise fatal disease.

## ATTR Cardiac Amyloidosis

Transthyretin (ATTR) amyloidosis is categorized into hereditary and wild-type amyloidosis on the basis of sequencing of the *TTR* gene. Wild-type ATTR cardiomyopathy (wtATTR-CM) is an under-recognized cause of restrictive cardiomyopathy and heart failure with preserved ejection fraction (HFpEF) (17, 18). Autopsy studies indicate that the prevalence of cardiac wtATTR amyloid is greater than previously thought with one particular autopsy study demonstrating deposits in ~25% of myocardial samples in patients aged 85 years and above (19, 20). Despite these studies, the true prevalence of wtATTR-CM remains unknown. wtATTR-CM is diagnosed in males between 25 and 50 fold more often than in females, and usually presents beyond the seventh decade of life although it may occasionally present in patients as young as 50 years (4, 21). Whilst the more frequent use of repurposed bone scintigraphy and CMR imaging have resulted in a noticeable rise in diagnoses in the past few years, the increasingly aged population is also likely to be contributing to the prevalence statistics of wtATTR-CM (4). Recent estimates suggest wtATTR-CM as the cause of 13–16% of cases of HFpEF in elderly patients (17). Clinically, wtATTR-CM typically presents with features of restrictive cardiomyopathy often preceded by carpal tunnel syndrome, lumbar canal stenosis and tendinopathies, in many cases up to 15 years prior (3, 21).

In contrast, patients with hATTR amyloidosis may present at a younger age with features consistent with cardiomyopathy, cardiac conduction disease, peripheral neuropathy and/or autonomic neuropathy. The clinical phenotype can consist of a mixture of both neuropathy and cardiomyopathy (22). There are over 130 pathogenic mutations of the *TTR* gene and although there is considerable overlap, there is a reasonably strong association between the specific mutation and the predominant clinical phenotype with certain mutations giving rise to predominant ATTR-CM, others predominant ATTR-PN, and the majority associated with a mixture of neuropathy and cardiomyopathy (23). One of the most important mutations is that encoding the p.V142I TTR variant (formerly known as V122I), which is carried by 3–4% of the Afro-Caribbean and African American population; an estimated 2 million mutation carriers in the US alone (3, 24). The disease penetrance associated with this mutation is thought to be low; however, the prevalence of hypertension and diabetes is high in this ethnic group such that misdiagnosis of hATTR-CM as hypertensive or uraemic cardiomyopathy is likely in a proportion (4, 24). Other relatively commonly encountered amyloidogenic mutations include those encoding the p.V50M and p.T80A TTR variants, which typically present at a younger age than wtATTR-CM with a combination of neuropathy and cardiomyopathy, or in the case of early-onset p.V50M with neuropathy in the absence of cardiomyopathy. Whilst amyloid polyneuropathy results in severe and progressive neurological disability and is associated with a significant disease burden, it is the ATTR-CM which has the greatest negative impact on prognosis, with a median survival in the absence of disease-modifying therapy, of 4–5 years from diagnosis (25). Given the impact of CA on prognosis in both AL and ATTR amyloidosis and the availability of new disease-modifying therapies, timely diagnosis using appropriate imaging techniques is essential to improving both quality of life and prognosis in these diseases.

## Electrocardiography

Electrocardiography is a widely accessible tool in both the community and hospital setting. Historically, the medical literature has characterized cardiac amyloidosis by low voltage QRS complexes. However, whilst low voltage QRS complexes coupled with evidence of LV thickening should correctly raise the suspicion infiltrative cardiomyopathy, the absence of low voltages by no means excludes CA. Indeed, the reported prevalence of low voltage QRS complexes varies considerably, ranging from 27 to 84% of AL CA patients in different studies (26). Low voltage QRS complexes are even less common in ATTR CA, with one particular study demonstrating that 44% of patients diagnosed with p.V142I-associated ATTRv CA had normal voltage QRS complexes, and a further 26% actually meeting voltage criteria for left ventricular hypertrophy (27). Therefore the absence of low QRS voltages on ECG should not prevent the assessing clinician from considering CA.

Cardiac conduction disease is a common finding in patients with ATTR CA, and it is not uncommon for pacemaker insertion to become necessary during the course of the disease. First degree heart block has utility in identifying patients at high risk of needing cardiac pacing (27). Pseudoinfarct patterns and poor R wave progression are also common ECG findings. Atrial arrhythmias, particularly atrial fibrillation are common in ATTR CA, likely reflecting the burden of atrial amyloid infiltration.

## Echocardiography

**Figure 1:**
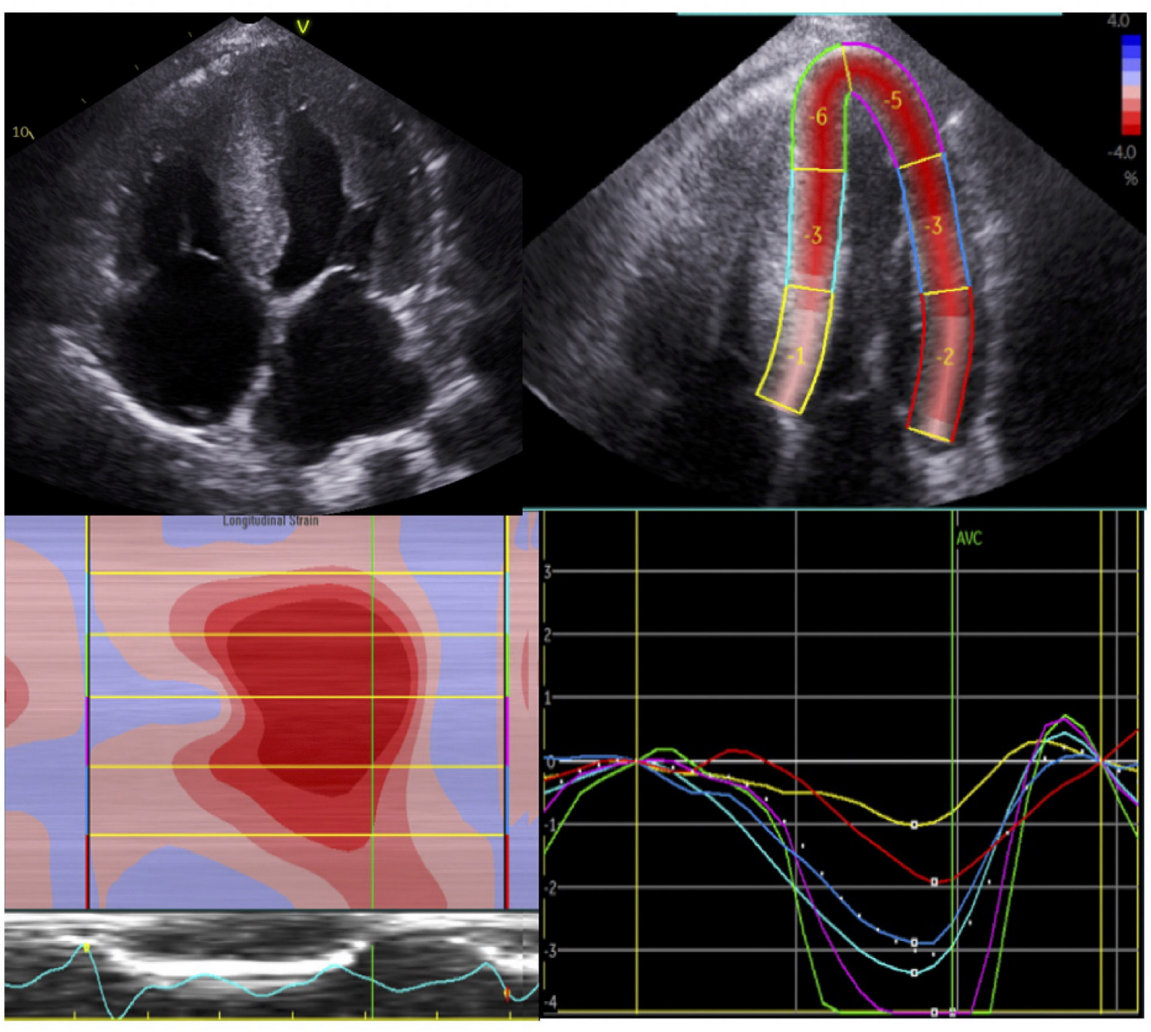
Echocardiographic findings in a patient with advanced transthyretin cardiomyopathy. Four chamber view demonstrating increased biventricular thickening with a “speckled” myocardium (top left panels) with 2D-strain using speckle tracking echocardiography in the same patient (top right panel). Peak systolic strain for individual myocardial segments in the four-chamber view panel and the strain curve samples in each of the corresponding colored myocardial segments panel can then generate a longitudinal strain map (bottom). With an estimated GLS of −4.3%, the characteristic basal to apical gradient of impaired longitudinal function is observed here.

Echocardiography is a widely accessible and risk-free first line imaging modality to assess patients with possible amyloid cardiomyopathy. The cardiac amyloid phenotype is characterized by left ventricular or biventricular thickening associated with a wall thickness >12 mm, although cardiac amyloidosis, especially of AL type, may be present in the absence of increased LV mass. A “speckled” appearance of the myocardium, with sparkling and thickened appearance of cardiac valves is described in CA, however this is neither sensitive nor specific. In CA, longitudinal function is usually affected before radial function, and therefore ejection fraction cannot be used as a reliable measure of ventricular function. Global longitudinal strain measurements derived from myocardial speckle tracking has emerged as a method to distinguish between amyloid cardiomyopathy and other causes of myocardial thickening such as hypertensive or hypertrophic cardiomyopathy (3, 4, 26). In CA two-dimensional strain mapping characteristically demonstrates a relative preservation of apical function leading to a bulls-eye pattern on strain plotting (Figure 1) (4). The relative differences in longitudinal strain in the basal and apical segments can be quantified into a ratio of LV apical and basal strain, which carries a poorer prognosis when apical sparing is seen (3, 4, 28).

Both mitral and tricuspid annular plane systolic excursion (MAPSE, TAPSE) have been proven to be prognostic indicators in CA (29, 30). The relatively early impairment of longitudinal function with preserved radial function, affecting predominantly basal segments over apical segments, is uncommon in other cardiomyopathies and is highly characteristic of CA.

Diastolic function is frequently severely impaired in patients with advanced disease with an E/e' which is often significantly elevated, reflecting diastolic dysfunction and raised filling pressures (31). Amyloid infiltration into the myocardium commonly affect the atria, causing atrial wall thickening, atrial dilatation (although severe dilatation is uncommon, probably reflecting the increased stiffness of the atrial wall), impaired ejection force and strain (32). A recent large scale study of 906 patients with ATTR-CM demonstrated that increased atrial stiffness was an independent predictor of poor prognosis. Additionally, there was significant impairment of the 3 phasic functional atrial components (atrial reservoir, conduit and contraction function); indeed 22% of patients in sinus rhythm with atrial electromechanical dissociation had absent atrial contraction. The extensive amyloid infiltration of the left atria on echocardiogram in this study was corroborated with histological analysis of explanted ATTR hearts (33).

Amyloid infiltration of the cardiac valves is usually associated with regurgitant physiology of the mitral and tricuspid valve. Chacko et al. demonstrated the association between significant mitral or tricuspid regurgitation and a poor prognosis in ATTR-CM. Coexistence of cardiac amyloidosis and aortic stenosis independently conferred a significant reduction in survival in patients with ATTR-CM (median survival 23 vs. 55 months) (31, 34). The severity of dysfunction measured by a range of echocardiographic parameters was also shown to differ substantially at the time of diagnosis between patients with three important ATTR-CM genotypes. Those with p.V142I-associated ATTR-CM had the worst echocardiographic parameters, followed by wtATTR-CM and finally p.T80A-associated ATTR CM, entirely consistent with the prognosis of these three conditions (33).

Both ventricular and atrial blood stasis have been demonstrated in studies to be substrate for intracardiac thrombi (35). Atrial electromechanical dissociation has been identified as a distinct clinical phenotype in ATTR-CM conferring a poorer prognosis, which merits discussion and consideration of anticoagulant therapy in such patients (33). In addition to the above, pleural and/or pericardial effusion are not uncommon findings in CA, especially in AL-CM (36). Whilst it is not possible to distinguish between AL-CM and ATTR-CM by echocardiography alone, certain features are seen more commonly in either type; namely a more symmetrical increase in LV wall thickness in AL-CM vs. an asymmetrical thickening in ATTR-CM. Up to 70% of ATTR-CM cases have a sigmoid septal morphology and there is septal curvature inversion in up to 30% of cases; these features are unusual in AL-CM (3, 36).

**Figure 2:**
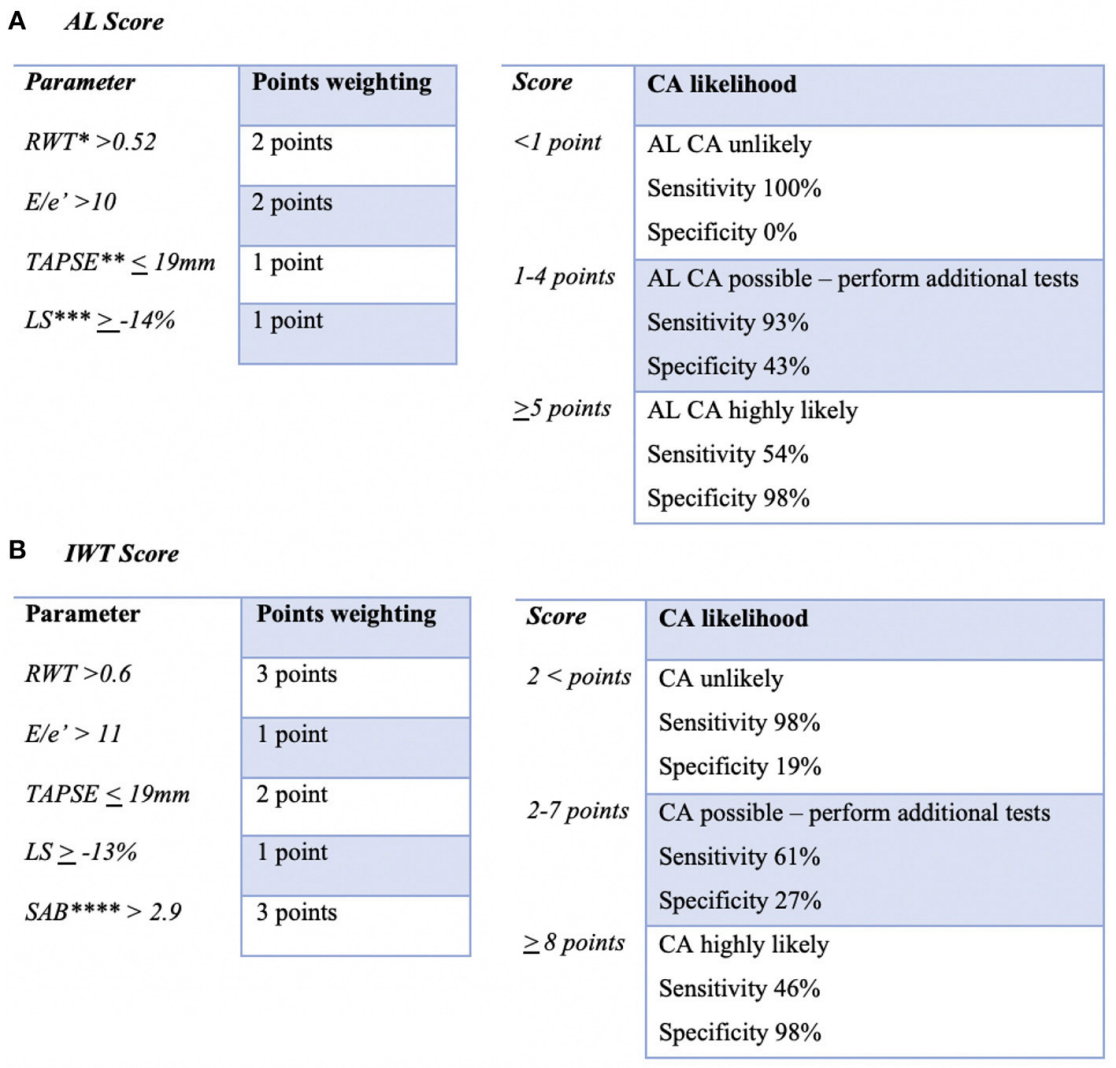
Adaptation of echocardiographic scoring system proposed by Boldrini et al. Two scoring systems were proposed for the evaluation of the possibility of cardiac amyloidosis depending on the clinical scenario. The first being confirming/excluding cardiac involvement in patients with systemic AL amyloidosis **(A)**, the second being confirming/excluding cardiac amyloidosis in patients with increased LV wall thickness (IWT) **(B)** (35). One could consider the variables used in this scoring systems as echocardiographic “red flags” that would raise suspicion of CA to the sonographer/scanner. *RWT, relative wall thickness; **TAPSE, tricuspid annular plane systolic excursion; ***LS, longitudinal strain; ****SAB, systolic apex to base ratio.

A recent study looked to develop a scoring system using specific echocardiographic parameters to identify patients who were likely to have cardiac amyloid. Boldrini et al. studied two cohorts of patients; one with systemic AL amyloidosis, and one with a hypertrophic phenotype. These two cohorts represented two key clinical scenarios; the first being a patient with systemic AL amyloidosis in whom cardiac involvement must be confirmed or excluded in order to guide chemotherapeutic decisions, and the second being a patient with a hypertrophic phenotype in whom CA must be confirmed or excluded as the cause. Relative wall thickness, longitudinal strain, TAPSE, E/e' and septal apical-to-base ratio were identified as variables that had diagnostic performance in identification of cardiac amyloid (see Figure 2). These variables had reasonable specificity for CA, and if significantly abnormal, could be considered as ‘red flags’ that should alert scanning clinicians to the possibility of CA. Because confirming or excluding CA has a significant impact on treatment strategy and prognosis, the authors proposed using highly sensitive and specific cut offs to exclude or confirm the diagnosis of CA. In patients with an intermediate probability of CA, use of second-level imaging modalities was encouraged (CMR, bone scintigraphy) as well as endomyocardial biopsy in order to confirm CA and distinguish between AL and ATTR types (37).

## Cardiac Magnetic Resonance

Cardiac magnetic resonance imaging has revolutionized the field of cardiac imaging, particularly in cardiac amyloidosis. CMR provides highly accurate, and detailed characterization of cardiac tissue and morphology, and is instrumental in distinguishing between cardiac amyloidosis and other hypertrophic phenocopies. The expansion of extracellular volume that results from amyloid fibril deposition within the myocardial extracellular space is accurately visualized using the administration of gadolinium-based contrast agent Gd-DTPA (gadolinium diethylenetriamine penta-acetic acid). “Late gadolinium enhancement” (LGE) on CMR gives rise to a characteristic, almost pathognomonic distribution; it is typically diffuse, subendocardial and/or transmural on CMR (36, 37). This is typically coupled with “abnormal gadolinium kinetics” whereby the gadolinium and the blood null at the same time. In the context of CA, gadolinium passively distributes in the expanded extracellular space created by amyloid fibrils, which gives rise to the aforementioned appearances. It has also been shown that the transmurality of LGE enhancement (i.e., from subendocardial through to transmural) is directly linked to disease severity and prognosis and reflects the underlying infiltrative process of CA (36, 38). However, the visualized LGE appearances on CMR are dependent on the assumption that there are areas of healthy and otherwise “normal” myocardium for comparison in remote segments. These areas of healthy remote myocardium may not always exist or be readily apparent to the CMR operator in cases of CA. This can lead to the human error of “nulling” abnormal myocardium as opposed to normal myocardium (37). Over the years, this drawback has been overcome with the development and adoption of phase sensitive image reconstruction (PSIR) which is a more reliable method as it overrides the need for operator chosen null points (36, 37). However, LGE does have disadvantages, most notably the risk of nephrogenic systemic fibrosis in those with impaired renal function (eGFR <30 mL/min). There are also studies demonstrating that although gadolinium is administered in a chelated form, tissue retention, specifically neurological tissue retention, of gadolinium contrast can occur in a dose dependent nature. The clinical consequences of this are not currently fully understood (39). It is not uncommon for patients with CA, particularly AL CA, to have concurrent renal disease, either as a result of direct renal infiltration of AL amyloid or as part of the cardio-renal syndrome from their cardiac failure. In these particular cases, the benefits of gadolinium contrast administration must be carefully balanced against the risks whilst closely involving the patient in these discussions and ensuring their informed consent before proceeding. Usually, if it is felt that a gadolinium contrast guided CMR would substantially change the patient's management by proving either the presence of AL or ATTR CM and thereby making them eligible for targeted therapy, the risk of nephrogenic systemic fibrosis is usually outweighed by the availability of prompt, targeted disease-modifying therapy.

Myocardial T1 mapping, a pixel-based reconstruction of measured longitudinal relaxation times, complements the use of LGE in the role of CMR in CA. In addition to its diagnostic utility, it can be used to monitor and track myocardial amyloid infiltration and therefore disease severity (40). In contrast to LGE, native myocardial T1 (a measure of T1 time before the administration of contrast) provides an objective quantitative measurement as opposed to a subjective qualitative one; this lends to its utility as a disease tracker. Native T1 provides similar diagnostic performance in both AL and ATTR CA, and is frequently found to be elevated in the early stages of CA prior to the development of biventricular thickening or detectable LGE (40, 41).

**Figure 3:**
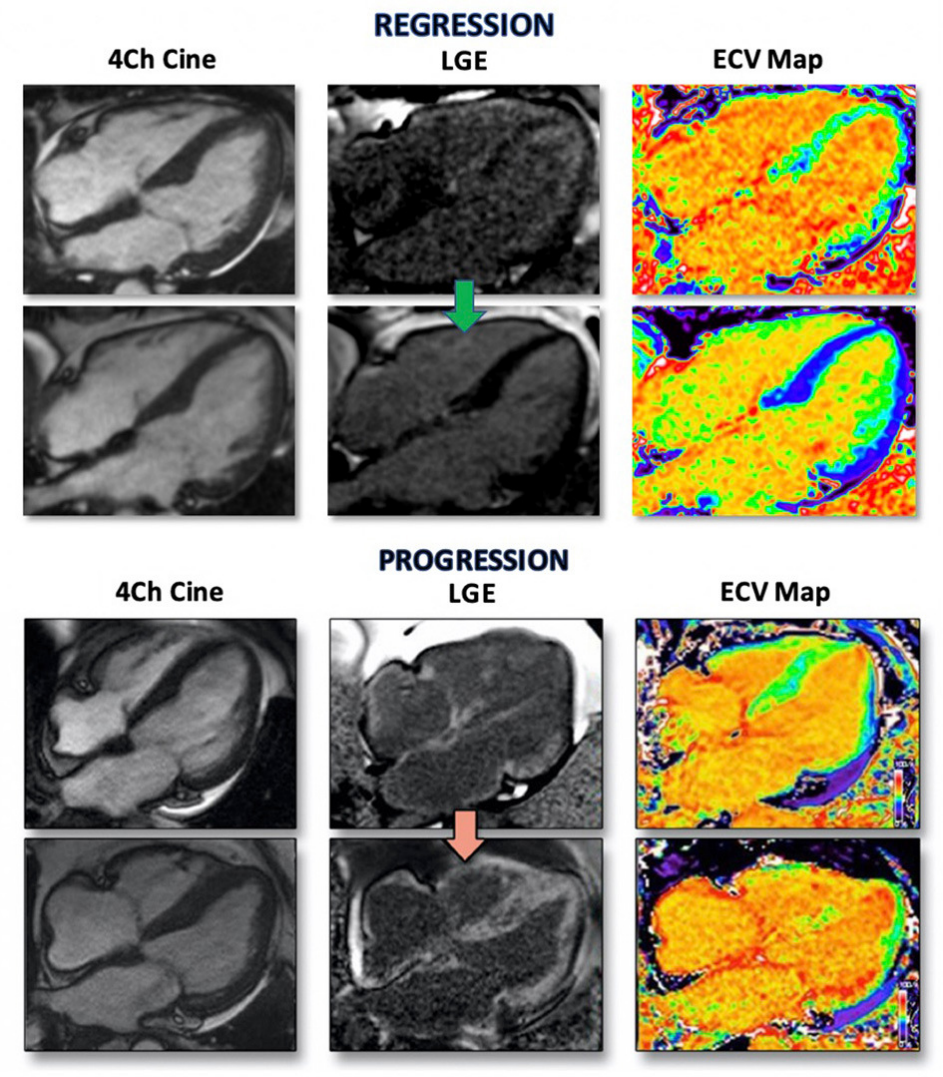
CMR images of two patients with AL CA regression and progression. Four chamber CINE image demonstrating characteristic biventricular thickening (left) with matched LGE images (middle) and ECV maps (right). Top panel is from a patient with a complete hematological response to chemotherapy treatment. Regression of cardiac amyloid is demonstrated by reduction in LV thickness, visualized LGE and normalization of ECV. Bottom panel is from a patient with disease progression reflected by increasing ventricular wall thickness, higher volume LGE and increased ECV (41).

Whilst T1 mapping is a sensitive marker for CA, it is a composite signal of both the extra and intracellular space. However, by administering gadolinium contrast, the ratio of pre and post contrast T1 maps can be used in conjunction with the patient's haematocrit level to isolate a signal to the extracellular space, and the extracellular volume (ECV) maps can be quantifiably measured (42). ECV maps are now the standard measurement technique for quantifying myocardial amyloid burden, and has demonstrated correlation with disease severity in both ATTR and AL CA (3, 42). ECV has validated utility in tracking changes over time, particularly in the context of monitoring response to treatment in AL CA (43) (Figure 3).

Chacko et al. recently demonstrated that ECV measurements during routine CMR can also quantify the burden of extracardiac amyloid, specifically splenic and hepatic amyloid in systemic AL amyloidosis patients (44). Not only does this provide important information to the reviewing clinician of the amyloid type, but it has potential to track extracardiac response to treatment. Prior to this, the only method to non-invasively investigate for extracardiac, namely hepatic and splenic amyloid, was serum amyloid P component scintigraphy (SAP scintigraphy) (45). Whilst this did provide an accurate visual assessment of the presence of visceral amyloid, it is only available in two centers globally. Being able to quantify splenic and hepatic amyloid via routine CMR ECV mapping therefore means it is now easier for clinicians to both diagnose disease, and track response to treatment more routinely and with a more accessible imaging modality.

T2 mapping serves to complement T1, LGE, and ECV methods in CMR and is a sensitive method to detect myocardial oedema found in a number of cardiac pathologies, both ischaemic and inflammatory. Examples would include acute myocardial infarctions, stress cardiomyopathies/Takotsubo's and myocarditis (46). T2 relaxation time is the constant governing the decay of transverse magnetization, and the proportional increase in T2 is larger than that found in T1 when free water content is increased within the myocardium (47). Modern developments have led to T2 mapping evolving into a quantifiable measurement, and therefore a potential tracker of disease progression and response to therapy (47). T2 mapping has been shown to be prognostically relevant in AL CA. Recent studies demonstrated that a cohort of untreated AL CA patients had higher T2 values and therefore myocardial oedema than treated AL and ATTR CA patients (13). The presence of myocardial oedema further confirms the fact that multiple pathophysiological processes and mechanisms of myocardial damage exist in AL CA.

In summary, CMR is a sensitive, accurate and reproducible imaging modality in the diagnosis and monitoring of patients with both AL and ATTR CA. It has also been shown to have utility in the quantification of extracardiac (hepatic and splenic) AL amyloid burden, which previously could only be assessed with SAP scintigraphy at two centers globally.

### Bone Scintigraphy

The use of ^99m^technetium-labeled-pyrophosphate (Tc-PYP) for bone scintigraphy and myocardial uptake in patients with proven CA was first demonstrated 1983 (48). However, the specific ligand responsible for tracer uptake in the myocardium of patients with CA remains unknown today. In 2005, bone scintigraphy using ^99m^Tc-3,3-Diphosphono-1-2-Propanodicarboxylic Acid (Tc-DPD) was shown by Rapezzi et al. to be highly sensitive for ATTR-CM and specific for CA generally (49). Subsequent work has provided greater clarity on the biological basis for the different “Perugini” grades of uptake reported in the original study (50).

**Figure 4:**
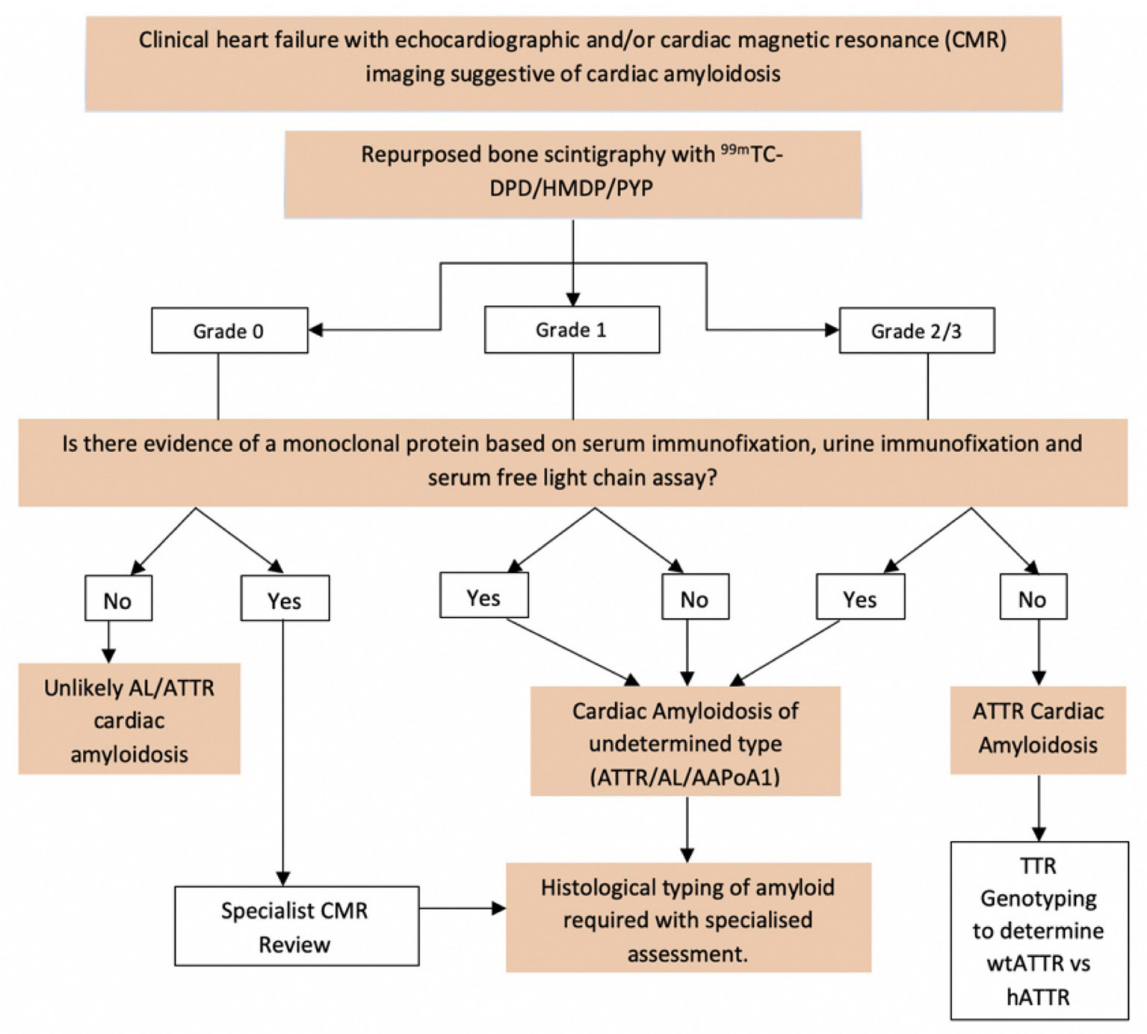
Non-invasive diagnostic algorithm and criteria for investigating patients with suspected CA. Echocardiographic features suggestive of CA include LV wall thickening, “speckled myocardium,” reduced global longitudinal strain with apical sparing and diastolic dysfunction. CMR features of CA include increased LV wall thickness and mass, elevated T1 values, subendocardial and/or transmural LGE in a diffuse circumferential pattern and elevated ECV measurements. In patients with a plasma cell dyscrasia and cardiac uptake on DPD, histological confirmation by way of cardiac biopsy is almost always required in order to confirm amyloid type (AL vs. ATTR) and guide appropriate therapeutic decisions. Equally, invasive histology is not required to confirm the diagnosis of ATTR CA should the appropriate imaging and biochemical criteria be met (Grade2/3 uptake on Tc-DPD, normal free light chains and immunofixation) (8).

An algorithm combining radionuclide scintigraphy (with DPD, PYP or HMDP) with echocardiography, CMR and biochemical investigations has enabled the diagnosis of ATTR amyloidosis to be established in the absence of a tissue biopsy in a substantial proportion of patients. In the absence of a monoclonal protein (by serum and urine immunofixation as well as by serum free light chain assay), a CMR and/or echocardiogram suggesting CA with a Perugini grade 2 or 3 radionuclide scan is diagnostic of ATTR-CM (8). However, it is important to note that the radionuclide scan alone is not specific for ATTR-CM since ~40% with AL-CM have a positive radionuclide scan (including Perugini grade 2 or 3 positivity in ~10%) and cardiac apolipoprotein A-I amyloidosis is also associated with low grade cardiac uptake on Tc-DPD scintigraphy (8, 51). Patients with a CMR or echocardiogram suggesting amyloid and grade 2 or 3 cardiac uptake on radionuclide scintigraphy who have a monoclonal protein by any of the aforementioned tests have a differential diagnosis of ATTR-CM and AL-CM. Subsequent histological confirmation of the amyloid type is required for a definitive diagnosis, often *via* an endomyocardial biopsy. The full diagnostic algorithm for CA is shown in Figure 4.

One important role of radionuclide scintigraphy is in excluding other cardiomyopathies in patients with biventricular thickening or LV hypertrophy. In patients with diffuse cardiac uptake on radionuclide scintigraphy one can be confident that CA is the cause of the aforementioned biventricular thickening. However, the converse is not true as radionuclide scintigraphy cannot be used to exclude CA, since up to 60% of AL-CA is associated with a negative radionuclide scan. Additionally, while radionuclide scintigraphy is extremely sensitive in ATTR-CA, rare hATTR variants such as S77Y and Y114C demonstrate less than expected uptake on DPD scintigraphy. Therefore should other imaging modalities (such as echocardiography and CMR) be strongly supportive of ATTR-CA in patients with the aforementioned TTR variants, CA cannot be excluded based on bone scintigraphy and must still be considered.

**Figure 5:**
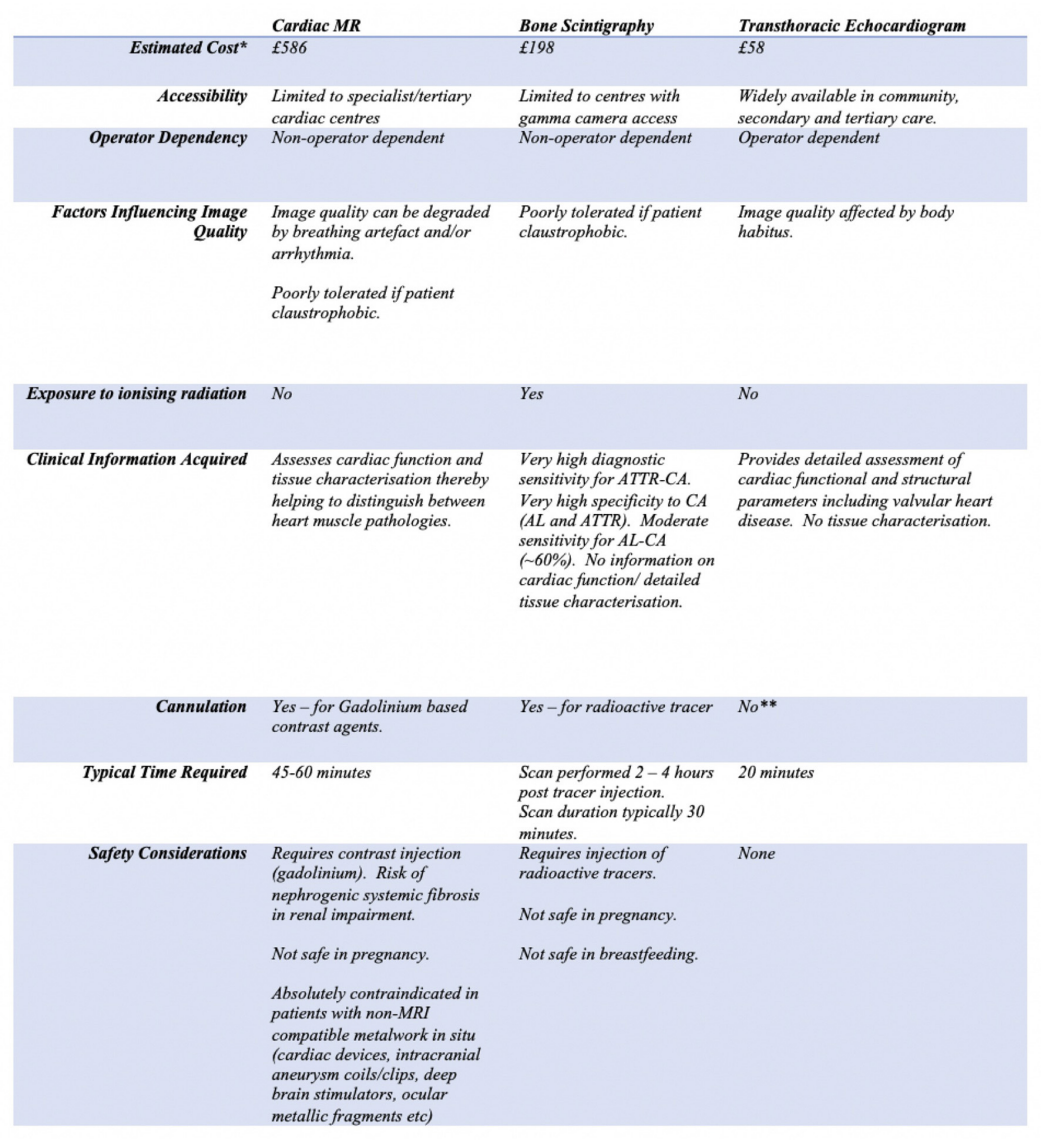
Table highlighting the individual limitations and strengths of cardiac magnetic resonance, bone scintigraphy and echocardiography in the context of assessing patients with cardiac amyloidosis (*costing estimates based on UK National Health Service Tariffs 2020/2021, estimate includes cost of reporting scans, **whilst cannulation is required for contrast echocardiography, this is not routinely indicated when assessing for CA).

The multimodality imaging advances highlighted above have allowed clinicians to diagnose and exclude CA, and in some cases, definitively determine the amyloid type without recourse to invasive endomyocardial biopsies whilst also permitting cardiac amyloid burden to be carefully tracked. Diagnosis of ATTR-CM can be reliably established without recourse to histology in ~70% of patients using these newer imaging tools which are increasingly accessible in most modern healthcare systems in combination with biochemical testing. CMR has proven to be a highly sensitive imaging modality in both AL-CM and ATTR-CM allowing for earlier diagnosis and subsequent successful therapeutic intervention in these diseases; which were historically treated with considerable nihilism due to the delays in diagnosis experienced by the vast majority of patients. Whilst individual imaging modalities have their own unique advantages, they also each have disadvantages (see Figure 5). Therefore, a multimodality approach with CMR, bone scintigraphy and echocardiography being used synergistically must be adopted in order to achieve the most accurate and comprehensive assessment of patients with CA.

## Current and Future Therapeutics

For many years, patients with systemic amyloidosis and in particular those with cardiac involvement, had few or no treatment options. Care mainly focused on supportive therapy and symptomatic relief, with no targeted therapeutics available to improve both quality and length of life. However, the past few years have seen landmark drug discoveries, particularly in the treatment of ATTR amyloidosis. Existing therapies are targeted at either reducing production of circulating TTR, or stabilizing circulating tetrameric TTR. Furthermore, novel therapeutics aimed at accelerating removal of existing amyloid are under development (3). Tafamidis, a TTR stabilizing agent, demonstrated a reduction in all-cause mortality and admissions to hospital in patients with ATTR-CM (52). Both inotersen and patisiran, an antisense-olgionucleotide and an RNA interference therapeutic, respectively, demonstrated stability or improvements in quality of life and neurological function in patients with hereditary ATTR amyloid neuropathy, with the latter suggesting improvement in ATTR-CM (9, 11). These and next generation disease-modifying therapeutics are also specifically being tested in ATTR-CM. Vutrisiran, a next generational agent developed from patisiran is currently being investigated in phase 3 clinical trials for patients with ATTR CA and neuropathy (NCT04153149 and NCT03759379). Vutrisiran is an RNA interference therapy similar to patisiran, but has the advantage of being administered infrequently and subcutaneously rather than intravenously every 3 weeks. Acoramadis, a next generation TTR stabilizing agent, is in phase III clinical trials in patients with ATTR-CM (NCT03860935). An alternative to the RNA interference technology used in current therapeutics is the clustered regularly interspaced short palindromic repeats and associated Cas9 endonuclease system (CRISPR-Cas9) to achieve *in vivo TTR* gene editing. ATTR amyloidosis is an ideal disease for testing this new therapeutic strategy since TTR has limited normal and specific function (namely; transport of thyroxine and vitamin A for which there is overlap in function with other proteins), the clinical benefit of TTR knockdown in the disease is established, and circulating TTR is entirely liver-derived. Interim findings from a first-in-human dose escalation study of the CRISPR-Cas9 therapeutic, NTLA-2001, were extremely encouraging with patients achieving up to 96% knockdown of circulating TTR concentration following a single infusion (53). Further studies of NTLA-2001, specifically in ATTR-CM, are anticipated.

Novel therapeutic targets have also been hypothesized for ATTR amyloidosis; in transgenic mouse models, knockout of specific complement pathway genes resulted in a significant increase in amyloid deposition suggesting that complement manipulation could be exploited as a future therapeutic strategy (54–57).

In AL amyloidosis, rapid suppression of the underlying clonal dyscrasia is the mainstay of treatment. Aggressive chemotherapy is the treatment of choice in this group of patients (1, 2). The preferred first line chemotherapy regimen depends on patient age and comorbidity as well as the extent and severity of organ involvement (58). Whilst high dose melphalan and autologous stem cell transplantation is one such option, a significant proportion of patients with AL amyloidosis are deemed unsuitable for this therapy due to age, renal impairment and end organ damage including advanced AL-CM (58), further emphasizing the critical importance of early diagnosis in a disease which progresses rapidly in the absence of intervention.

## Conclusion

Cardiac amyloidosis has, until recently, been considered to be uniformly progressive and rapidly fatal. However, numerous advances in both diagnosis and therapy in recent years have transformed the landscape for patients suffering with CA. Imaging advances in CMR and echocardiography have helped to develop our understanding of disease mechanisms, and when used appropriately, serve as accurate, widely available and sensitive diagnostic tools in assessing patients with suspected CA. Radionuclide scintigraphy with DPD, PYP or HMDP plays a vital role in complementing the above imaging modalities which together, can exclude alternative cardiac pathologies with a high degree of sensitivity. With promising therapeutics available commercially, both through healthcare systems and within clinical trials, early diagnosis facilitated by the advanced multimodality imaging techniques highlighted in this review will permit therapeutic intervention at an earlier stage of the disease course in patients with CA and likely impact dramatically on clinical outcomes. Furthermore, the ability to accurately evaluate treatment responses using multimodality imaging which will enable the plethora of novel therapies to be individually tailored, hails a better future for patients with CA.

## Author Contributions

YR performed the literature review, manuscript preparation, and draft editing. RP and MF performed draft editing. JG performed final draft review and editing. All authors contributed to the article and approved the submitted version.

## Conflict of Interest

JG is an advisory board member and consults for Alnylam Pharmaceuticals, Ionis, Intellia Therapeutics, and Eidos. MF consults for Ionis, Alnylam Pharmaceuticals, Pfizer, Eidos, and Intellia Therapeutics. MF is funded from a British Heart Foundation Research Fellowship (FS/18/21/33447). MF holds research grants from Pfizer and Eidos. The remaining authors declare that the research was conducted in the absence of any commercial or financial relationships that could be construed as a potential conflict of interest.

## Publisher's Note

All claims expressed in this article are solely those of the authors and do not necessarily represent those of their affiliated organizations, or those of the publisher, the editors and the reviewers. Any product that may be evaluated in this article, or claim that may be made by its manufacturer, is not guaranteed or endorsed by the publisher.
